# 

**Published:** 1918-10-05

**Authors:** 


### THE HOSPITAL

*THE MODERN NEWSPAPER OF ADMINISTRATIVE MEDICINE AND INSTITUTIONAL LIFE*

INCLUDING

ADMINISTRATION, NATIONAL INSURANCE, HEALTH

AND

THE INTERESTS OF ALL INSTITUTIONAL WORKERS

OCTOBER 1918 TO MARCH 1919

VOLUME LXV

SURGEON GENERAL'S

241424

LIBRARY

LONDON

PUBLISHED BY THE SCIENTIFIC PRESS (LIMITED), AT *THE HOSPITAL* BUILDING

28 & 29 SOUTHAMPTON STREET, STRAND, W.C. 2.

MDCCCCXIX

AS THE CONDITION OF THIS VOLUME
WOULD NOT PERMIT SEWING, IT WAS
TREATED WITH A STRONG, DURABLE
ADHESIVE ESPECIALLY APPLIED TO
ASSURE HARD WEAR AND USE.

THIS NEW TYPE OF ADHESIVE IS
GUARANTEED BY
HERTZBERG-NEW METHOD, INC.

W/ NO69X

### INDEX TO VOL. LXV.

Aberdeen Nurses' Club and Midwives' Association, 316
Adami, Professor J. G., 421
Addison, Dr., 4, 13, 128
Addresses of Patients' Friends, 490
Adolescent, Teaching the, 467
Advertising, The Tricks of, 44; Can an Institution Advertise too Much? 260; Health and Remedies, 507; A Doctor on, 514
Affluent Working-man, A Doctor's Warning to the, 86
After the Allies' Triumphant Victory, 182
Air Ministry, Allegations against the, 397
Air-raids, 382, 439
Alcohol Consumption in Military Hospitals, 124, Nov. 23, vi
Alexandra Day, 95; At Bournemouth, 106
Allen, Mr. Richard, 64
Allotment Holders' Harvest, 43
Almeric Paget Massage Club, 501
Alsop, Miss, 314
Amalgamated Society of Drug and Chemical Workers, 367
Ambulances, 327
America (*see* United States)
Anæsthetics, German Operations without, 242
Anderson, Miss A. M., 338
Annual Meetings, Feb. 15, viii, 577
Annuities for Hospitals, 215
Anthrax Wool, Sterilisating of, 513
Anti-Toxin instead of Sleep, 326
"Appliance"? What is an, 108
**APPOINTMENTS:** Medical, Dec. 28, iii, 428, 578; Nursing, Oct. 5, iv, 40, Oct. 19, iv, 82, 102, 123, Nov. 16, iv, 190, 208, Dec. 14, iv, 256, 297, 322, Jan. 18, iv, 394, Feb. 8, vi, Feb. 18, viii, 462, March 1, vi, 508 533, March 22, vi
Archangel, Photographs of, 280
Armistice, The, 131. 139
Army Education of Military Hospitals, 347
Army Hospitals' Accounts, 561
Army Nurses, 56; Thanks to, 185, 291
Artificial Limbs, 42, 174, 340
Association des Orphelius de la Guerre, Oct. 5, iv
Association of Hospital Officers, 209
Asylum, Visiting Committees, Conference, 178; Officers' Strikes, 188; Proposed Central Body, 371; Marriage of Medical Officers, 507
Atkinson, Miss E., 186
Australia, Bush Nursing, 56; Notes, 137
Austrian Wounded, 173, 183
Auxiliary Hospitals, Closing of, 204, 223
Axworthy, Lance-Corporal C. W., 303

Baby Week (*see* Infant Welfare)
Balfour, Lady Frances, 98
Balkans, Collapse in the, and the Drug Supply, 6
Banbury, Money-raising Scheme, 102; Horton Infirmary, 244
Bangor, New Infirmary for, 301
Barnes, Dr. Stanley, 194
Barr, Sir James, and the Medical Profession, 1
Barrasford Sanatorium, 540
Barton, Miss, 165
Basil Blackwood Day Nursery, 244
Battle, The Site of, 5
Bazy, L., 195
Becher, Dame E. H., 476
Belfast, Service at, 242
Belfast Union Infirmary, 56
Belgravia War Hospital Supply Depôt, 360
Bell, Dr. E. S., 398
Belmont Road Military Auxiliary Hospital, 17
Beri-beri, Prevention of, 560
Bermondsey Guardians, 152, 153, 175, 290, 540
Berry, Miss, 316
Bethnal Green, The Grave Scandal of, 479, 539
Bexhill, Nursing and Health Work, 380
Biernacki, Dr., 55
Binyon, Mr. Laurence, 46
Bird, Miss, 350
Birkenhead, Lack of Nurses at, 151
Birmingham, Bishop of, 467
Birmingham, Board of Guardians, 39. 500; A Lift for Charities, 195; M.O. to the Pensions Hospital, 347; Medical Schools, 440
Blakesley, Major H. J., 420
Blind, The, French Soldiers, 64; American Soldiers, 64; Home for Babies, 532
Blinds and Influenza, 340
Board of Education, 39, 283
Boyd, Col. F. H., Feb. 1, vi
Bradford Housing Scheme, 302
Brampton Board of Guardians, 303
"Brave Poor Things," 268

**BOOK WORLD:**
Bruce: Materia Medica and Therapeutics, 474
Country Rector's Wife, A.: Flourless Puddings and their Sauces, 92
Davies: Forty New Poems, 298
Deane: Gymnastic Treatment, 298
Delafield: The War Workers, 286
Denman: The Discharged Consumptive Soldier, 298
Dentists' Register, The, 6
Duval: War Wounds of the Lung, 11
Eliason: First Aid in Emergencies, 12
Elliott: Glaucoma, 11
Falconer: Arthrometer, or The Measurement of the Movements of Joints, 376
Ferguson: Thyrea and Other Sonnets, 12
Forster: How to Become a Woman Doctor, 92
Guest: Patriotism and Plenty, 298; Casting Out Fear, 376
Hinton: Rats and Mice as Enemies of Mankind, 546
Hurst: Medical Diseases of the War, 9
Keith: Clinical Case-Taking, 376
Kleen: Massage and Medical Gymnastics, 285
Macfarlane: The Economic Basis of an Enduring Peace, 474
Maher: A Sister of a Certain Soldier, 285
Maternity and Child Welfare, 59
Medical Annual, The, 6
Medical Register The, 6
Mercier: Crime and Criminality, 473
"Padre," The: 50,000 miles on a Hospital Ship, 199
Robinson: National Reconstruction, 11
Runting: First Aid for Foot Troubles, 298
St. Johnston: The Lau Islands (Fiji), Their Fairy Tales and Folklore, 91
Scharlieb: Notes on Venereal Diseases for Nurses and Midwives, 12
Seekings: Manuals of Health. The Baby, 201
Sheba: Vaccines and Sera in Military and Civilian Practice, 200
Short: An Index of Prognosis, 199
Stevens: A Review of the American Hospital, 179
Stopes; Wise Parenthood: A Sequel to "Married Love," 200
Strachey: Eminent Victorians, 49
Wallace: Surgery at a Casualty Clearing Station, 375

BOOK WORLD.—*continued.*
Weatherley: A Plea for the Insane, 200
White and Martin's Genito-Urinal Surgery and Venereal Diseases, 200
Wilson: The Hearts of Man, 474
Wood: The Soldier's First Aid, 201
Wrightson; An Enquiry into the Analytical Mechanism of the Internal Ear, 92
Boshill, Nurse, Death of, 243, 338
Bourne, Cardinal, 174
Bradford Board of Guardians, 86, 174, Proposed New Infirmary, 87; Hospital and Convalescent Fund, 233
Bray Court, Maidenhead, 5
Brigade Hospital of the Order of St. John, 76
Brighton, Pavilion Hospital, 34, 295; Hospital Sunday, 303
Bristol Board of Guardians, 302
British Columbia, Medical Men in, Nov. 23, vi
British Fire Prevention Committee, The, 122
British Hospitals After the War, 61
British Hospitals' Association, 40, 61, 79
British Hospitals under Bombardment, 382
British Medical Association: The Law of the Land, 62; Absence of Statesmanship, 178; Meeting, 559
British Psychological Society, 488
Broken Pledge and Its Consequences, A, 4
Brown, Mr. E. Lionel, 489
Brown, Dr. R. C. 281
Brown, Sir R. D., 86
Browne, Dame, Sydney, 292, 313, 478
Burtchaell, Sir C. H., 301
**BUREAU OF INFORMATION, OUR** (*see* Institutional Worker—the Supplement to THE HOSPITAL)
Burnett, Sir Napier, 511
Burns, Mr. Emile. 467
Bursey, Dr. Elspet, 153
Byles, Miss E. M., 55

Cadbury Bros., Ltd., 501
Caduceus, 114
Cæsarian Section, a plea for, 517
Calcutta, Health Visitors in, 440
California House, 316
Camberwell Borough Council, 64, 350, 467
Camberwell Infirmary, 410, 458
Canada: The Invalided Soldier, 6, 45; University Education for Nurses, 99
Cancer of the Tongue and Tobacco, 324
Cannings, Mr. R. B., 489
Canterbury, Archbishop of, 209
Cape Provincial Council, 141
Cardiff: Prince of Wales' Hospital, 124, 164, 562; The German Fleet Surrender, 193; Maternity Committee, 430; University College, 559
Carmarthen, The Laundry Deadlock, 106
Carruthers, Miss, 34
Carter, Mrs. 478
Carter, Mr. Brudenell, 93
Casualty, An Unusual Battle, 529
Catholic Nursing Guild, 174
Cavell, Miss Edith, 34, 206; Commemorative Service at Brussels, 243; The Chaplain's Story, 272; Homes of Rest, 314; Proposed Removal of Body, 364.
Central Association for Mentally Defective. 87
Central Council for District Nursing, 476
Central Committee for the State Registration of Nurses, 140
Central Midwives Board, 476
Central Midwives Board, Scotland, 165, 290, 478
Cerebro-Spinal Fever, 578
Certified Occupation List, Staffs and the, 27
Chairman, The Rarer Type of, 152
Chancellor, Mr. Beresford, 217
Chaplain, The Dismissal of a, 281
Charlton, Sister, 574
Cheshunt Smallpox Hospital, 532
Children of Soldiers, 513
Child's Fear of the Dark, A, 280
Cholera in Petrograd, 16
Christ Church Guardians, 409
Christmas, 184; Nurses' Holidays, 242; Hospital Appeals, 253; Games and Decorations, 260; Leave for Patients, 268; In Hospitals, 293, 317
Cinderella, 415
Cinemas, 130; Influenza Epidemic, Nov. 23, iv
City Guardians and St. Bartholomew's, 153
Clare Hall Sanatorium, 148
Classification by Institution, 174
Claygate Village Nursing Association, 269
Cleansing of Persons Act, 559
Clemenceau, Dr., 193
Clifford, Miss Mary, 359
Clifton, Alderman G., 489
Clinical Memoranda for Practitioners, 29
Clinical Thermometers, 88, Nov. 16, iv
Club for Nurses, Proposed New, 551
Coal, Certificates for, 173
Cobb, Sir Cyril, 64
Cocaine Inquest, 379
**COLLEGE OF NURSING:** 20, 33, 36, 40, 54, Oct. 19, iv, 73, 76, 97, 99, 119, 123, 139, 140, 141, 182, 183, 184, 202, 224, 226, Dec. 14, iv, 274, 289, 290, 314, 332, 335, 339, 356, 358, 360, 377, 382, 383, 408, Feb. 15, viii, 455, 456, 459, 475, 476, 477, 478, 485, 515, 521, 523, 550, 552, 571, 572, 576; Demobilisation Bureau, 164, 336; Irish Branch, 35
CENTRES:
Aberdeen, 35, 322; Birmingham, 123, 170, 382; Brighton, 504, 523; March 29, vi; Bristol, March 29, vi; Cambridge, 17, 170, 362, March 22, iv, 550; Derby, 484, 504; Dundee, 35; Edinburgh, 170, 188, 290, 322, March 29, vi; Glasgow, Jan. 18, vi, 382; Liverpool, 99, 102, 119, 362, 504, March 22, iv; Leeds, 56, 123, 484; London, 16, Jan. 18, vi; 335, 382, 484, March 29, vi; Manchester, 16, 35, 76, 141, 412; Northumberland, 123; Nottingham, March 22, iv; Sheffield, 56; Yorkshire, 123, 141, 170, 412
Collie, Sir John, 181, 397
Collins, Dr. Beale, 4
Combination, Nurses in, 521
Constantinople, Nurses' Voyage to, 228
Control of a Hospital, The, 558
Convalescent Home for Seamen, 105
Convalescent Homes, The Fees of, 303
Convalescent Soldiers, Light Occupations for, Supp. Nov. 23, Nov. 30, Dec. 7, 14; Lectures for Officers, 195
Cooper, Dr. P. R., 373
Co-operation, The Spirit of, 203
Coroners, Where are the, 87
Cottage Hospitals as War Memorials, 360
Cottage Hospital Matrons, For, 66
Cottingham, Miss H. M., 119
County Councils' Association, The, 87
Cowlin, Miss Gertrude, 20, 36, 336
Cream, The Supply of, 281
Croydon Mothers' and Infants' Welfare, 260
Crusades, The End of All, 85
Cummins, Miss, 430

Damascus, The Capture of, 75
Dartford: American Hospital, 340, 409
Davies, Major David, 128
Davies, Mr. W., 348
Dawson Ideal, The, 86
Defence of the Realm Act, 163
**DEMOBILISATION:** Nurses, Nov. 16, iv, 337, 524, 550, 578; Hospitals, 184, 223; Mental Defectives, 234; Red Cross Nurses, 356; Doctors, 325, 371, 485, 578; Dental Surgeons, 325; Private Auxiliary Hospitals, 364; Red Cross, 374.
Democracy, The National Limits of, 4
**DENTAL:** Army Service, 146; Public Clinics, 280; Release of Army Dental Surgeons, 325; Treatment for Mothers, 441; The Nation's Teeth, 465; Dentists Act, Parliamentary Report, 509; Nurses and Dental Clinics, 511; Shortage of Dentists, 530; Gratuity to Officers, 556
Deptford Health Centre, 502
Derby: New Infirmary, 184
Development of English Spas, The, 422
Dickson, Mrs. H. C., 420
**DISCHARGED AND DISABLED SOLDIERS:** 70, 87, 160; Discharged Officers, 219; Tuberculosis, 303, 530; Medical Treatment, 507
**DISPENSING** (*see also* Drugs): Child Welfare, 45; Commissioned Rank and Army Pharmacists, 45; New Salaries, 46; In U.S.A., 95; Examination Changes, 175; Praise for Italian Pharmacists, 260; Dispensing Medicines in the Army, 347; Pharmacist Examination Changes, 327; Trade Unionism, 367; Fewer Registered Pharmacists, 422; Pharmacy in the Seventies and Eighties, Feb. 15, iv; The British Pharmacopœia, 465; Certified Assistants to Apothecaries, 578
District Nursing, Apathy and, 369
Doctors' Estates, 39
Doctors' Political Meeting, The, 31
Domestic Service, 378, 415
Dover: Red Cross Sale, 215
**DRUGS** (*see also* Dispensing), The Week's Market In, 6, 28, 46, 66, 88, 108, 130, 154, 176 196, 218, 236, 261, 282, 304, 328, 350, 370, 400, 422, 442, 468, 490, 514, 540
A Cruel Hoax, 28; After the War, 230; Drug-Taking in the West End, 236, 379; Tampering with Prescriptions, 326; Sale of Cocaine and Morphine, 465; The Law as to Sales, 470; Needed Restrictions, 488
Duchess's Retort, The, 82
Dunmow Guardians, 529
Durham County Council, 369
Durham County Midwives' Federation, 369
Dutch Colleges of Nursing, 400
Dutch Medical Tradition, 88

Easterbrook, Dr., 2
East Riding County Council, 457
Economies in Heating, Lighting, and Accessories, Supp. Dec. 28
Edinburgh: Thanksgiving Service, 185; Royal Visit, 203; Clubs for Nurses, 290
**EDITOR'S LETTER-BOX:**
Aberdeen Shows the Way, 19
Atkinson, the Late Miss, 188
Birmingham, Influenza Crisis at, 187
Boots or No Boots, 57
Children's Hospital, The Case of the, 411, 433, 460
Children's Nurse's View of Registration, A, 434, 481
College of Nursing, What we Expect from the, 273, 287, 321, 381, 459
Communion Cup and Infection, 229
Co-operative Appeals, 187, 381
Demobilisation, Payments for Nurses, 434
Discharged Soldiers and Their Outlook, 288, 242
"Do-Nothings" and the College, 525
Dublin Meeting, The, 525, 553
Each British Woman to be Stirred Up, 169
Factory Nurse's Salary, The, 503, 526, 575
Front Line Club Suggested, 207
Fulham Infirmary Nursing League, A, 78
Health of the Nation, The, 411

EDITOR'S LETTER-BOX.—*continued.*
Ierne: Taken to Task, 38; Some Questions Answered, 101; An appreciation, 246
Indian Mission and the Hospital, An, 554
Irish Nurses' Tribute Fund, The, 122
Is Every Woman a Nurse? 145, 169
Is Nursing a Profession? 459, 553
Lady Roberts' Field Glass Fund, 276
Leeds General Infirmary, 207
London Hospital Nurses, 19, 37, 57, 77, 321
M.A.B. Nurses, The Shortage of, 58
Madness, The Treatment of Incipient, 57
Matron, The Efficient, 381
Medical Men, Grave Injustices to, 411
Moral or Legal Responsibility, 525
Night Duty and Patients' Sufferings, 245, 287, 342, 364, 412
Nurse Alice Pettit's Articulation, 187
Nursing Homes; Criticism with Suggestions, 434
Nursing Sister, The Exclusive Title of, 481
Overpaid and Underpaid Hospital Nurses, 245
Patient's Protest, A, 208, 245
Position of Nurses in Great Britain, The, 526
Private Patients, Right to Send to District Hospitals, 229
Ray, Mrs., Presentation to, 228
Repatriation of the Serbians, The, 144
Royal British Nurses' Association, 481
St. Bartholomew's New Nurses' Home, 575
Scottish Nurses in London, 246
Should the Nurse Articulate? 208, 245
"Sick House" for Private Nurses, A, 412
State Medical Service, A, 169, 187, 229, 341
Strike in Lloyd's Tobacco Factory, The, 273
Tradition or Progress, 77
Venereal Diseases, 309, 503
Edmonton Board of Guardians, 107, 267
Education: Higher Education of Nurses, 20; Physical Training, 188
Eggs and Bacon for Infirmary Staffs, 65
Eggs, The Price of, 281
Eight-Hour Day for Nurses, An, Supp. Jan. 11, Jan. 18, Feb. 1
Electricity and Medicine, 568
Elsie Inglis Unit, 26, 34; Memorial Fund, 246
Endowing a Bed, the Cost of, Supp. Mar. 29
Engineers and Disease Prevention, 154
Entertainments Tax, The, 43
Epidemic and the Public, The, 149
Equipment, Surplus, 537
Ethics of Controversy, 459
Evans, Sir L. Worthington, 397
Exercising Apparatus, An Original, Supp. Jan. 25
Expectations and Disappointments, 437

Face Masks, Should we Wear, 399
Factories, Welfare Work in, 148; Medical Inspection, 556
Factory Surgeon on the Housing Question, A, 281
Farming, Institutional, 151
Farquhar, Viscount, 259
Fenwickism and How to Fight It, 202
Fever Hospital, A Small, Supp. Dec. 14
Fever Nurses' Association, 55, 430
Field, Mrs. E. M., 500
Fiji, Curiosities of Native Treatment in, 333, 405, 445, 495
Finance of Transition, 153
Fire Drill to Fuel Drill, From, 5
Fisher, Sir Scott, 85
Fleas and Influenza, 340
Flying Machines and Flying Men, 231
**FOOD:** Maternity Rations, 6; Ministry of Food Recipes, 18; Government Pamphlet, 56; Inspection and Standard Ships, 105; The World's Outlook, 244; Records of Rationed Foods, Supp. Jan. 4, 1919; Mr. Hoover, 338; No Reduction in Prices, 380; Invalid Rations, 420; Easing of Rationing Scheme, 440; Work of Inspectors, 447; Diet and Influenza, 548
Forecast, A Remarkable, 132
Forging Ahead, 32
Forthcoming Events, Oct. 5, iv., 40, Oct. 19, iv., 78, 102, Nov. 23, vi., 312, 344, 364, 394, 416, 530, March 22, vi, March 29, vi
Foster Mothers, 502
Fourth Southern General Hospital, The, 282, 292, 320
Fragment, A, 189
France, Pressure in Military Hospitals, 316
Fraser, Sir Thomas, 86
From Across the Seas, 436, 554
Fryatt, Capt., 364
Fuel, Additional Allowances, 96
Fuel and Lighting Economies, Supp. Oct. 12, 19, 26, Nov. 2, 16
Fulham Infirmary, 123
Future and the Nation's Choice, The, 151

Gas Warfare, Can Treaties Abolish, 191
Gazette of 3rd London General Hospital, 66
Gahan, Rev. H. S. T., 206
Games, A Moralisation of, 321
Garden Produce and Preserves for the Hospitals, Supp. March 1
General Practitioner, Self-Help for the, 367
**GERMANY:** Wounded Left Behind, 76; Scientists Barred, 94; Deterioration of Women, 99; Beware of Prussian Legislation, 128; Surrender of Fleet, 193; Railway System, 282
Ghoul Type of Nurse, The, 455
Gibson, Mr. J., 348
Gibson, Miss Jessie, 573
**GIFTS AND BEQUESTS,** 60, 144, 252, 364, 393, 508, 534
Gillett, Mr. F. H., 108
Glasgow: The Shakespeare Hospital, 87; Post Graduation Study at, 154; The Royal Infirmary, 193; Club for Nurses, 290; King's Fund for, 301; Gift to University, 340
Glories of the Profession and Non-Combatants, 558
Glycerin, 174, 196
Goodwin, Sir John, 301
Gosford, Countess of, 56
Gradual Teaching and Medical Reconstruction, 113
Graham, Mr. Howgrave, 63
Greenwich Metropolitan Auxiliary Hospital, 4
Grey, Lord, 53
Guest, Capt. L. Haden, 195, 302
Gum Arabic in Stock, 441

Hall, Miss C. M., 33
Hamer, Dr., 113, 155
Hammersmith Borough Council, 143
Hammersmith Public Health Committee, 36
Hampstead Council of Social Welfare, 184
Hanwell Mental Hospital, 5
Hardy, Thomas, 3
Hastings War Memorial, 380
Hay-Box, The, 532
Health Visitors, The Salaries of, 66, 173, Feb. 1, vi; V.A.D. Workers, 269; Lectures for, 337, 342; Influenza, 368
Heather-Bigg, Miss, 36
Heine-medin Disease, 147
Heliotherapy, Developments in, 284
Heredity and Health, 155; In Hospital Life, 537
Hertfordshire County Council, 142, 523
Hertfordshire County Nursing Association, 55
Higher Education: Nurses' Need, 183; Effect on Salaries, 313, 377
Hill, Miss Amy, Oct. 19, iv
Hill, Dr. A. Bostock, 512
Hincks, Mr. W. E., 40
Hocking, Mr. F. A., 367
Hodge, Mr. John, 260
Holland, State Prisoners in, 107; Private Nursing of Lunatics, 107
Holmes, Mr. Charles, 468
Home Helps, Training, 218, 369
Home Hospitals' Association, 63
**HONOURS:** 300
Albert Medal, 408
French Honours, 56, 127
Mentions, 291, 378, 409
Military Cross, 17, 74
Military Medal, 409
New Year's, 279, 291, 301
Order of the British Empire, 313, 335, 343, 364, 462, 549
Peace Investiture, 164
Royal Red Cross, 17, 74, 97, 118, 140, 164, 183, 242, 267, 268, 291, 431, 456, 477, 499, 524, 549, 550, 571, 572
Hooley, Mr., 27
Hope, Professor E. W., 489
**HOSPITAL AND INSTITUTIONAL NEWS,** 3, 25, 43, 63, 85, 105, 127, 151, 173, 193, 215, 233, 259, 279, 301, 325, 347, 367, 397, 419, 439, 465, 487, 511, 537, 559
**HOSPITAL AND INSTITUTIONAL RESULTS:** 60, 555
Hospitals and the Local Public, 512
**HOSPITAL ARCHITECTURE AND CONSTRUCTION:** 179 (*see* also The New Era)
Hospital Clinical Services and the New Age, 171
Hospital Construction (*see* New Era, The)
Hospital Extensions, are they Urgent? 5
**HOSPITALS, INSTITUTIONS, ETC.:—** (War, Temporary and Auxiliary Hospitals. *See under* Individual Names)
METROPOLITAN:
Alexandra Hospital for Children, 382
All Saints Hospital, 153
Anti-Vivisection Hospital, 108
Battersea General Hospital, 305
Belgrave Hospital for Children, 65
British Home and Hospital for Incurables, 153
Brompton Hospital, 108
Central London Ophthalmic Hospital, 506
Charing Cross Hospital, Oct. 5, iv, 34, 36, 118, 352, 360, 382, 456, 476
Chelsea Hospital for Women, 153, 320, 532
Christ's Hospital, 316
City of London Hospital for Diseases of the Chest, 153
City of London Lying-In Hospital, 489, 506
"Dreadnought" (*see* Seamen's Hospital)
East London Hospital for Children, 153, 165, 271
Elizabeth Garrett Anderson Hospital, 43, 540, 551, 577
Evelina Hospital, 153
Fitzroy House, 561
Foundling Hospital, 233
French Hospital, 259
Great Northern Central Hospital, Oct. 5, iv, 54, 119, 122, 127, 151, 175, 302, 314, 327, 400, 577
Guy's Hospital, 105, 106, Nov. 16, iv, 293, 326, 419, 467, 545
Hospital for Epilepsy and Paralysis, 63, 153
Ilford Emergency Hospital, 142
Italian Hospital, 382
King's College Hospital, 105, 151, 153, 227, 303, 317, 417, 421, 438, 487, 551
London Homœopathic Hospital, 183, 382
London Hospital, 14, 38, 124, Nov. 23, vi, 211, 294, 362, 448, 451, 452, 456, 483, 505, 521, 522, 545
Metropolitan Convalescent Institution, 468
Metropolitan Hospital, 153
Middlesex Hospital, 55, 295, 359
Miller General Hospital, 233
Mount Vernon Hospital, 430
Mothers' Hospital, Clapton, 44
National Hospital for the Paralysed and Epileptic, 5, 127, 153, 382, 577
Orphan Working School, 60

HOSPITALS, ETC.—*continued.*
Paddington Green Children's Hospital, 193
Plumstead Children's Hospital, 66
Queen Charlotte's Hospital, 362
Queen Mary's Hospital, 75, 506
Queen's Hospital for Children, 367, 489
Royal Dental Hospital, 246
Royal Free Hospital, 34, 60, 215, 228
Royal General Dispensary, 439
Royal Hospital for Diseases of the Chest, 577
Royal Hospital for Incurables, 246, 261, Dec. 28, iii, 360
Royal London Ophthalmic Hospital, 530
Royal Surgical Aid Society, 233
St. Bartholomew's Hospital, 98, 153, 233, 294, 326, 382, Feb. 1, vi, 429, 545, 572, 574
St. Columba's Hospital, 236
St. Mary's Hospital, 269, 317, 340, 367, 488
St. Mary's Hospital, Plaistow, 476, 506
St. Thomas's Hospital, 34, 61, 129, 186, 209, 210, 545
Samaritan Free Hospital, 572
Seamen's Hospital, Greenwich, 105, 127, 317, 352, 506, 522, 531
Sunderland, Royal Infirmary, 175
Sydenham, Hospital for Sick Children, 513, 555
University College Hospital, Nov. 16, iv, 276
West London Hospital, 205
Westminster Hospital, 396, 417, 438
Willesden, The New Hospital, 233, 398
PROVINCIAL, SCOTTISH AND IRISH:
Abercarn, Goole Hospital, 490
Aberdeen, Children's Hospital, Oct. 5, iv, 35, 320; Royal Asylum, 86, 439, 490
Alton, Crippled Children's Hospital, 322
Ashton, Lake Hospital, 45
Barnsley, Beckett Hospital, 145, 562, 572, March 29, vi
Bath, Royal United Hospital, 56, 184
Beccles, New Hospital, 398
Bedford County Hospital, 349, 359, 490
Belfast, Royal Victoria Hospital, 98, 108
Birkenhead and Wirral Children's Hospital, 420
Birmingham General Hospital, 154, 369, 539; Queen's Hospital, 194; Nervous Diseases Hospital, 195; St. Chad's Hospital, 538
Blackburn and East Lancashire Royal Infirmary, 439
Bournemouth, Royal Victoria and West Hants Hospital, 120
Bournemouth, Royal Victoria Hospital, 194, 290, 420
Bradford, St. Luke's Hospital, 369; Eye and Ear Hospital, 514, 537; Royal Infirmary, 537; Children's Hospital, 537
Brighton, Women's Hospital, 151
Bristol, General Infirmary, 227, 410, 514; Royal Infirmary, 281; Hospital for Children, 537
Broxbourne Cottage Hospital, 349
Bury St. Edmunds, West Suffolk Hospital, 142, 196, 467
Buxt·d, St. Mary's Home, 380
Cambridge, Addenbrooke's Hospital, 216
Cardiff, King Edward VII. Hospital, 43, 54, 85, 151, 260, 378, 396, 419, 522, 523, 559; Royal Hamadryad Seamen's Hospital, 538
Carnarvon Cottage Hospital, 301
Carnwath, Captain T., 399
Cheltenham, General Hospital, 281
Colne Cottage Hospital, 216
Croydon, Whitgift's Hospital, 439
Derby, Royal Infirmary, 195; Hospital for Women, 561
Dewsbury General Infirmary, 398
Dublin, Mercer's Hospital, 233
Durham, Sherburn Hospital, 176
Edinburgh Royal Infirmary, 86, 141; Royal Hospital for Incurables, 500
Enfield Cottage Hospital, 397, 555, 558
Essex County Hospital, 318
Exeter Royal Devon Hospital, 399, 409, 466, 511
Gateshead Children's Hospital, 537

HOSPITALS, ETC.—*continued.*
Glasgow Royal Infirmary, 60, 105, 215, 336, 439; Western Infirmary, 105, 215, 227, 291; Victoria Infirmary, 105, 327; Royal Maternity Hospital, 259; Royal Hospital for Sick Children, 397
Gravesend Hospital, 382
Halesowen Cottage Hospital, 420
Harrow Maternity Hospital, 501
Hastings, East Sussex Hospital, 380; Buchanan Hospital, 380
High Wycombe, War Memorial Hospital, 350
Huddersfield, Royal Infirmary, 87
Ipswich, East Suffolk and Ipswich Hospital, 120, 368
Lancaster Infirmary, 369
Leamington, Warneford Hospital, 439
Leeds, General Infirmary, 186, 422, 514; Maternity Hospital, 399
Leicester, Royal Infirmary, 100, 130, 295, 355, 420, 489, 562
Liverpool, Royal Infirmary, 60, 295, 512, 574; Country Hospital for Children, 60; Royal Southern Hospital, 303
Maidstone, West Kent Hospital, 501, 555, 577
Manchester, Ancoats Hospital, 364, 398, 456, 551, 574; Eye Hospital, 400
Mansfield and District Hospital, 105, 523
Merthyr General Hospital, 562
Middlesbrough, North Ormsby Hospital, 439
Mitcham Hospital, 18
Newport, Royal Gwent Hospital, 538
Newcastle-on-Tyne, Royal Infirmary, 109, 457, 552
Norfolk and Norwich Hospital, 51, 319, 349, 539
Northampton General Hospital, 186, 319, 367, 378, 420
North Lonsdale Hospital, 26
Nottingham General Hospital, 35, 487, 522
Oswestry Cottage Hospital, 432
Paisley, Royal Alexandra Infirmary, 60
Papworth Hall Tuberculosis Colony, 27
Peterborough Hospital, 26
Pontypool and District Hospital, 25, Jan. 18, vi, 489
Portsmouth, Royal Hospital, 233, 537
Preston, Royal Infirmary, 319, 348, 522, 573
Reading, Royal Berkshire Hospital, 173
Richmond Hospital, 64
Ross Cottage Hospital, 422
Salisbury Infirmary, 297
Selby Cottage Hospital, 490
Sheerness Cottage Hospital, 129
Sheffield, Royal Infirmary, 76, 511; Royal Hospital, 511
Sidcup, Queen's Hospital, 204
Stockport, The General Infirmary, 336
Stoke-on-Trent, North Staffs Infirmary, 26, 319, 349
Stone Cottage Hospital, 216
Swansea, Royal Gwent Hospital, 327; General and Eye Hospital, 431
Tarbert Cottage Hospital, 128
Teddington Cottage Hospital, 511
Torquay, Ockenden Convalescent Home, 555
Tranmere Military and Civil Hospital, 151
Truro, Royal Cornwall Infirmary, 326
Tunstall Cottage Hospital, 537
Walsall General Hospital, 176
Warneford and Leamington Hospital, 26
Watford, London Orphan Asylum, Oct. 5, iv
Wigan, Royal Albert Edward Infirmary, 320
Winchester, Royal Hampshire County Hospital, 196
Winsley Sanatorium, 322
Wolverhampton and Staffordshire General Hospital, 60, 194, 439
York, County Hospital, 205, 234, 466
COLONIAL, U.S.A., &c.:
Hobart General Hospital, 554
Jerusalem Eye Hospital, 217, 560
Melbourne Hospital, 205
New York, Mount Sinai Hospital, Dec. 28, iii

HOSPITALS, ETC.—*continued.*
Sydney, Prince Alfred Hospital, 436, 540
Uganda, C.M.S. Missionary Hospital, Numirambe, 34
Hospital Needs and Hospital Appeals, 253
Hospital Saturday Fund, 3, 25, 123, 246, 303, 497
Hospital Ships: The *Aquitania*, 165; White Paper Issued, 193
Hospital Sunday Fund, 108, 212, 262, 349, Feb. 1, vi
**HOSPITAL WEAKNESS AND HOSPITAL STRENGTH**, 515
Hospitals' Future and Medical Practice, The, 41
Hostel for Patients' Friends, A, 87
Household Department, A Year's Work in a Hospital, Supp. March 1
Hostel for Wounded Soldiers, Nov. 23, vi
Houseley, Sister L. A., 379
Housing Problem, The, 161, 281, 303; A Royal Scheme, 325, 410; In Bethnal Green, 479
Hull, Friendly Societies' Conference, 102
Hospital Supplies Depôt, 204; Farm Colony for Consumptives, 255
Human Incineration, 180
**HUMAN TEMPERAMENTS**, 221, 567
Humidity and Health, 329
Hurst Sunshine Home Hospital, 151
Hygiene of Modern Armies in the Field, The, 351

Ierne's Criticisms and Apologia, 454
Imperial War Museum, 34
Incomes of Medical Men, Feb. 1, vi
India, Influenza in, 205
Indian Medical Service, The, 8
Industrial Fatigue, Research Board, 270; Study of, 332
Inefficients, The Percentage of Military, 352
**INFANT WELFARE AND MATERNITY:**
Maternity Rations, 6; Schools for Mothers, 39; Leeds, 45; Mortality and the Use of Cots, 65; Additional Fuel Allowances, 96; Powers Under New Act, 130, 148; In Durham, 153; Lectures, 162; Winchester, 196; Mrs. Irving, 260; Tilam and Codishead, 270; Breast Feeding, 337; Hotel for Babies, 368; Bethnal Green Centre, 379; Portsmouth, 399; Co-ordination, 409; Work in Wales, 432; Greenwich, 457; Medical Training, 492; Incubators, 532; National Baby Week, 534
Influenza and Immunity, 331
**INFLUENZA EPIDEMIC, THE**: 83, 68, 74, 98, 124, 149, 157, 160, 177, Nov. 23, vi, 186, 194, 196, 205, 215, 235, 261, 267, 268, 303, 304, 305, 340, 349, 368, 378, 379, 410, 421, 427, 441, 466, 478, 481, 500, 510, 519, 523, 524, 538, 548, 578
Ilford, Children's Hospital for, 301
Illegitimacy, Fallacies about, 368
Indian Doctors, A Tribute to, 400
Infirmaries, War Work of the, 218
Insane (*see* Mental)
Institute of Hygiene, 94, 168, 194
"Institute of Natality," 458
**INSTITUTIONAL FACTS AND FIGURES** (*see* The Institutional Worker—the Supplement to the Hospital)
Institutional Library (*see* Book World)
Inter-Allied Fellowship of Medicine, 559
Invalid Rations, 420
Ireland, Ministry of Health Bill, 466; Hospitals and State Control, 541
Irving, Mrs., 260
Italy: Signorino G. Vigano, 88; Red Cross in, 127, 270

Jam at Tea-time, A Little, 45
Jam-making in Hospitals, 74, 118, 140
Jews and the Jerusalem Eye Hospital, 217
Jezreel, 3
Joel, Mr. S. B., 173

Johncock, Sister E., 315
Johnson, Mr. T. Fielding, Jan. 18, vi
Jones, Col. G. A., 369
Jones, Sir Robert Armstrong, 71
Joynt, Dr. I. W., 328
Jury, Hospital Patients Form a, 63

Kensington Infirmary, 314
Kent County Council, 370
Kerr, Mr. Glenton, 367
Kerr, Dr., 127, 215
Keyes, Sir Roger, 487
King, Dr. Truby, 228
**KING, THE** Speech to American Soldiers, 63; The Throne and the Press, 63; Great Northern Central Hospital, 151; At Edinburgh, 203
King Edward's Hospital Fund, 147, 247, 257, 308, 546, March 22, iv
Kingston Nursing Association, 524

Ladies' Committee, The, Supp. March 8
Lambeth Guardians, 44, 164, 194, 280, 282, 397, 490
Lambeth Infirmary, 315
Lane, Sir Arbuthnot, 419
Law Courts, Sleeping in the, 234
Law, Miss E., 118
Lay Managers of Hospitals, 477
Leaflet Competition, 54, 225, 414
League of Mercy, The, 259, 275
League of Nations, 53
Leeds: Doctors in Church, 328; Health Committee, 457; Poor-Law Infirmary, 572
Legacies, A Good Week for, 105
Legacy of Military Control, The, 561
**LEGAL NOTES FOR INSTITUTIONAL WORKERS,** 506
Leicester: War Hospital, 166; Bequest for University, 488
Lewisham Military Hospital, 305
Lice and Public Health, 537
Lice-borne Diseases, Prevention of, 47
Life of a Nurse, The, 414, 435, 503
Lighterman's Thank-Offering, A, 106
Lighting in Wards, Indirect, 512
Limbles, Prince of Wales Hospital, 349
Limbless Soldiers, The Problem of, 466
Limerick Asylum, 457
Lincoln District Nursing Association, 315
Liverpool Health Committee, 422
Lloyd, Sir Francis 43
Lloyd's Tobacco Factory, The Strike In, 216
Local Government Board, 83, 152, 217, 228, Dec. 14, vi, 235, 243, 304, 359, Feb. 1 vi, 431, 481
Lombardini, A., 461
London Ambulance Column, The, 315
London Association of Medical Women, 367
London, Bishop of, 141
London County and Westminster and Parr's Bank, 342, Feb. 8, vi
London County Council, 105, 106, 113, 218
London Medical Missionary Association, 400
London School of Economics, 340
London University, 444
Londoners Hospital Beds Threatened, 417, 446
London's Health, 113, 155
Luckes, Miss E. C., the late, 448, 451, 452, 456, 483, 521, 522
Lunacy (*see* Mental)
Lying-in Homes Under the Maternity Act, Supp. Feb. 8, Feb. 15
Lyle, Mr., 573
Lyth, J. C., 237

McCreath, Sister, 360
McKay, Mr. James, 327
McKay, Miss Tait, 292
McKenzie, Mr. F. A., 22
Madness (*see* also Mental), The Treatment of Incipient, 2
Maidstone Poor-Law Infirmary, 551
Malaria, 421, 563
Manchester: New Hospital for Disabled Men, 87; Hospital Saturday Fund, 260; Sick Poor Nursing Institution, 410; Hope Military Hospital, 512
Manners, Mr. J. Hartley, 28
March Nursing Association, 166
Marriage, Health Certificates, 173; Earlier, 442
Marshall, Sir Horace, 173
Marylebone Infirmary, 314
**MASSAGE AND MASSEUSES:** In Military Hospitals, 146; Protest Against War Office Action, 501, 551; Navy and Army Masseuses, 530
Masks, 519
Maternity (*see* Infant Welfare)
Matron, The Post of, 116, 407
Matrons' and Sisters' Appointments (*see* Appointments—Nursing)
**MATRONS' AND SISTERS' DEPARTMENT,** 15, 33, 53, 73, 97, 116, 139, 163, 183, 203, 241, 267, 289, 313, 335, 357, 377, 429, 455, 475, 499, 521, 549, 571
Matrons-in-Chief: Luncheon to, 74, 118; Military and Overseas, 117
Maurice, Col. G. T. K., 109, 133, 197
Medical Curriculum, Shortening the, 4
Medical Fellowship, A, 346
Medical Impostors, 554
Medical Mission Exhibition, 216
Medical Officers of Health, Publicity and, 217
Medical Parliamentary Committee, The, 395, 439
Medical Practice, Influences of War on, 125
Medical Profession as a National Service, The, 104
Medical Reconstruction and Graduate Teaching, 213
Medical Research Committee, 146
Medical Society of London, 48
Medical Student, The Better Educated, 328
Medical Training, Which is it to be? 67
Medicinal Preparations and Substitutes, 232
Medicine and Politics, 299
Medicine and the Individual, 535
Medicine, Morals and manifestoes, 277
Medico-Political Union, The, 311
Melbourne, Nurses Overworked in, 205
**MENTAL:** Certified Occupation List, 28; In Holland, 107; Defectives and Demobilisation, 234; Conference at Guildhall, 347; Two Generations of Lunacy Legislation, 469; Military Occupation of Hospitals, 530
Mercier, Dr., 367
Methylated Spirits, Women and, 348
Metropolitan Asylums Board, 40, 130, 141, Nov. 16, iv, 217, Feb. 1, vi, 512
Metropolitan Hospital Sunday Fund (*see* Hospital Sunday Fund)
Midland Cottage Hospital, A New, 26
**MIDWIVES:** The Midwives Bill, 70; Conference, 165; Medical Treatment by, 218; Local Government Board, 359; Guarantee for, 369; Association for Promoting Training, 432; Mile-stone on the Road to Registration, 335; Derbyshire, 478
Military Hospitals: Position of Disabled, 242; Closing of, 302
Military Versus Medical Titles, 126
Milk: Increased Price, 18; London, M.O.H.'s Report, 64; Commandeered for Infants, 66; The Pure Milk Problem, 220; Death-Dealing, 421; Nottingham, 441; Public Supply, 467
Milk Bill in a Small Fever Hospital, The, Supp. Oct. 12
Milligan, Sir William, 328
**MINISTRY OF HEALTH:** 43, 94, 100: What the Bill Must *Not* be, 103; The New Act, 146; Teaching Hospitals, 159; Need for Organisation, 192; Women's Council, 242, 501; Institutional Officers, 261, Dec. 28, iii; Wigmore Hall Meeting, 401; Nurses' Pay, 413; Lady Rhondda, 458; The Bill, 463, 556; Ireland, 466; A Pamphlet, 497; The Next Step, 535; Guardians of the Poor, 518; Research Committee, 543
Ministry of Labour, 98
Ministry of Munitions, 532
Ministry of National Service, 27, 466
Ministry of Pensions, 5, 160, 223, 278, 370, 397, 398
Ministry of Reconstruction, 4, 13, 128, 338, 432
"Modern Hospital," The, 478
Molony, Sister, 431
Monk, Miss, 521, 522
Monmouthshire County Council, 5
Moore, Mr. F. W., 512
Morgan, Mr. Tom, 562
Morris, Mr. E. W., 14, 159
Morris, Sir Henry, 21
Morris, William, 26
Morris, Rev. J., 261
Mossley Hill, The Americans at, 4
Motherhood (*see* Infant Welfare)
Mould, Mr. William, 348
Municipal Hospitals, The Growth of, 561
Municipal Salaries and Duties, 46
Munition Workers, 206
Mysterious Disease, A, 304

Nairne, Sir Perceval, 327
Narcotics, The Popular Medical Uses of, 345
Natal, Training of Probationers, 502
National Association for Prevention of Consumption, 108
National Association of Trade Union Approved Societies, 442
National Birth-rate Commission, 467
National Council of Women, 475, 476
National Council for Combating Venereal Disease, 215
National Food Reform Association, 432
**NATIONAL HEALTH INSURANCE:** Certificates in Poor-Law Institutions, 44; War Bonuses and Medical Benefit, 124; Six Years of Panel Practice, 237; Panel Doctors, 556
National Health Society, 228
National Housing and Town-Planning Council, 410
National League of Health, 368
National Mission, 268
National Relief Fund, 174, 513
National Stamina Lowered? Is the, 181
National Thanksgiving and How to Show it, 258
National Utility Poultry Society, 281
Nation's Fund for Nurses (*see* College of Nursing)
**NAVY:** Medical Service, 487; Nursing Sisters, 507; Notes of a Surgeon, 547; Gratuities to Medical Officers, 578
Nerve-Strain and Heredity, 566
Netley, Welsh National Hospital, 562
Neurasthenics, 206
Newcastle Cathedral Nurses, 552
New End Military Hospital, 327
New Era, The, 354, 404, 480, 527, 569
Newman, Sir George, 41, 205, 283
Newsholme, Sir Arthur, Feb. 1, vi
New Zealand: A Business Inspector for Hospitals, 25; Hospital Board's Inquiry, 436; Registration Act Examinations, 502; Wellington Hospital Board, 574
Nightingale Legend, The, 49
Northampton Red Cross Work, 204
North Riding Nursing Association, 501
Nosocomia Lodge, 327
Notifiable Diseases, The New, 447
Nurses' Co-operation, 551
Nurse's Day and Future Outlook, The, 96, 121, 138
Nurses' Father Confessor, The, 152
Nurses' Missionary League, 18
Nurses' Note-Book, The, Supp. Nov. 30
Nurses' Registration Bill, 573

Nurses' Resettlement Bureau, 524
Nurse Training in Small Hospitals, 241
Nurses Without a Voice, 499
Nursing Homes, A Test for, 561
Nursing Profession and Nurses, The, 72, 428, 520
Nursing Reforms, The Nurse's Part in, 163
"Nursing Sister," Title of, 458
Nutt, Miss, 552

Obelisk, Be Careful of the, 26
**OBITUARY:** Dr. W. C. Woodhead, 26; Mr. Howgrave Graham, 63; Miss K. S. Stewart, 74; Sir Scott Fisher, 85; Dame Agnes Weston, 85; Mr. Brudenell Carter, 93; Miss A. B. Aked, Miss H. M. Cottingham, 119; Dr. John McCaw, 152; Mr. T. Robinson, 175; Miss L. Atkinson, 186; Sir Ernest Tritton, 279; Mr. William Mould, 348; Mr. W. F. Chalkley, 349; Prince John, 357; Miss Mary Clifford, 359; Col. J. A. Jones, 369; Dr. E. S. Bell, 398; Miss Luckes, 448; Mr. S. H. Rouguette, 487; Miss Jessie Gibson, 573
Offen, Mr. C. R. W., 26
Ogilvie, Dr. George, 259
Okey, Miss, 457
On Titular and other Decorations, 300
Order of the British Empire (*see* Honours)
Organisation, The Need for, 192
Out-Patient Records, Supp. March 29
Overlapping, The Organisation of, 421
Overseas Club and Patriotic League. Oct 5, iv

Panel Practice, Six Years of, 237
Parliament (*see also* Medical Parliamentary Committee): The Medical Profession in, 21, 31; Women in, 75, 98
**PARLIAMENT AND THE PROFESSION,** 70, 101, 124, 146, Nov. 23, vi, 188, March 1, vi, 507, 530, 556, 578
Parliament and the Universities, 171
Parliament, Doctors in, 23
**PASSING EVENTS AND TOPICS,** Oct. 5, iv, 39, 101, 122, 246, Dec. 28, iii, 340, 364, Feb. 1, vi, 532
Patent Medicines, 530
Pauperism, Decrease in, 338
Paying Patients, 569
Peace (*see also* Armistice), 139; Nursing Service in the Dawn of, 143; Appeals, 153; Paris Conference, 242; A Woman's Idea of, 267
**PENSIONS:** For Nurses in Military Service, 16, 556; The Permanence of, 115; Tuberculosis, 129; King Edward's Hospital Fund Report, 546
Percival, Mr. G. H., 367
Personalia, Dec. 28, iii, 508
Pharmaceutical Society, 327
Pharmacists (*see* Dispensing)
Physiognomy, On, 114
Pike, Miss A. G., 477
Politeness to Patients, 429
Poor-Law Infirmary Matrons' Association, 120
**POOR-LAW** (*see also* Local Government Board): Patient's Donation, A, 43; Politesse à la, 65; A Woman's Long Service, 350; Gardens, 399; Paying Patients, 400; The Approaching End of Boards of Guardians, 518
Porter, The Cottage Hospital, 88
Ports and the Nation's Health, 489
Portsmouth: A Municipal Hospital for, 233; Board of Guardians, 281; Infant Welfare, 399
Potatoes, 118, 140
Practitioners, A Public Duty to, 542
Prescriptions, The Forging of, 236
Presentations, 233
Preventive Medicine, 421, 545
Priestly, Dr. Joseph, 280
Prince of Wales, 203, 325
Prince of Wales's Fund (*see* National Relief Fund)
Prince of Wales's Hospital, Marylebone, 146
Prince John, Death of, 357
Princess Louise, 118
Princess Mary, 185, 204, 227, 243
Printers' Pension Fund for War Orphans, Dec. 28, iii
Prisoners in Holland, 107
Probation, The Reconstruction of, 570
Prohibition in the U.S.A., 403
Pro Patria Day Nursery, 552
Public Health (*see also* London's Health), 39, 59; Public Opinion, 83, 94; Work for Women, 414; Reports, 736; Bournemouth, 526; League of Nurses, 533
Publicity, The New, 129
Public Pharmacists' Associations, 442
Pulpit, Medical Officers in the, 513

**QUEEN, THE,** 27, 73, 203, 204, 226, 379, 539
Queen Alexandra, 51, 74, 127, 294
Queen Alexandra's Hospital for Officers, 303, 573
Queen Alexandra's Imperial Nursing Service, 17, 98, 476, 550, 573
Queen Alexandra's Naval Nursing Service, 556
Queen Mary's Hostels for Nurses, 476
Queen Mary's Needlework Guild, 340, 458
Queen Victoria's Jubilee Institute, 206, 522
Queen's Needlework Fund, 267

Rahere, Tomb of, 233
Ranyard Nurses, 206
Ray, Miss, 227
Rea, Mr. Leonard, 26
Reading in Bed, On, 516
Recent Official Publications, Oct. 5, iv, 102, March 1, vi
Reconstruction (*see also* Ministry of Reconstruction), The Call for, 289
Reconstruction of the Professions, The, 12
Reconstruction and the Voluntary Hospitals, 237
**RED CROSS** Our Day, 25, 75, 87, 127; Stonehenge, 26; Shortage of Beds, 54; Nurse Recruits, 56; Central Work Rooms 56; History of the Society, 71; Queen's Gift, 73; In Italy, 127, 270; Sale of Trees, 130; War Office Control, 168; Convalescent Officers, 195; Demobilisation of Hospitals, 204, 223; Sham Nurse, 206; Sale of Pearls, 270; The Red Cross Diarist, 279; Future of Working Parties, 291; Plymouth, 316; Ambulances for the People, 327; Demobilised Nurses, 356; Gold and Silver Tributes, 340; The Future of Hospitals, 370; Demobilisation, 374; Peace Time Work, Feb. 15, viii, 468, 476, 477; An Untapped Reservoir, 490
Registration of Nurses (*see* College of Nursing and Royal British Nurses' Association)
Reigate Guardians, 76
Relieving Officer's Dilemma, 195
Religious Nursing Sisters, 165
Research, The Advancement of Medicine by 323; Public Money and, 566; A Landmark, 402; Freedom in, 543
Reticence, The Cultivation of, 549
Rhodes, The Knight Hospitallers of, 528
Richardson, Mrs. T. E., 244
Roberts, Mr. G. Q., 210, 237
Ross, Sir Ronald, 421
Rouen: Scottish Hospital, 574
**ROUND THE HOSPITALS,** 16, 33, 54, 73, 92, 118, 139, 163, 183, 203, 226, 242, 267, 289, 313, 335, 357, 407, 377, 429, 455, 476, 499, 521, 549, 571
Rouguette, Mr. S. H., 487
Routh, Dr. A., 360
Roux, Dr., 161
Row, Miss A., 165, 271
Royal Air Force Medical Service, The, 7, 137, 145
Royal Army Medical Corps, 325; Prisoners of War Care Committee, 364
Royal British Nurses' Association, Nov. 16, iv, 167, 360, 459, 551
Royal National Pension Fund, 75
Royal Sanitary Institute, 40, 102, 148, 337
Royal Statistical Society, 518
Rural Population, The, 68
Russia, The Red Cross In, 86
Ruthin Workhouse Infirmary, 560
Rye: Mrs Jameson's Hospital, 291; Proposed War Memorial, 336

St. Bartholomew the Great, 234
St. Dunstan's Hostel, 153
St. John of Jerusalem, Order of, 502
St. Johnston, T. B., 333, 405, 495
St. Pancras Guardians, 431
**SALARIES, SISTERS AND NURSES,** 14, 32; Royal Free Hospitals, 34; Charing Cross Hospital, 34; Great Northern Central Hospital, 54; Queen Mary's Hospital, 75; Reigate Guardians, 76; M.A.B., 141; Norton Infirmary, Banbury, 244; Great Northern Central Hospital, 314; College of Nursing, 358; Bedford County Hospital, 359; Effect of Higher Education, 377; Royal Devon Hospital, 409; West Kent Hospital, 501, Seamen's Hospital, 522; Forest Hospital, Mansfield, 523; St. Bartholomew's, Beckett Hospital, Barnsley, City Hospital, Leeds, 572
Sales of Hospital Equipment, 398
Salvation Army, 22, 44
Sanatoria (*see* Tuberculosis)
Sanitary Organisation of Armies in the Field, 544
Sara, Miss, 142
School Doctors' and Nurses' Troubles, Feb. 1, vi
Science and the Prevention and Cure of Disease, 24
Scotland, Association for the Promotion of the Registration of Nurses in, 336
Scottish Blinded Soldiers' Hospital, 185
Scottish Women's Hospitals, 120, 292
Scurvy, Prevention of, 560
Serving the King's Men, 22
Sheffield: Chair of Anatomy, 340
Shell-Shock and Suggestion Treatment, 566
Shepherd, Mr. G. D., 540
Should the Nurse Articulate?, 162
Skinner, Mr. H., 367
Sleep: Shortage, 337; The Gift of, 363
Smallpox: Treatment of Contacts, 106; Camberwell, 467; In 1918, 556
Smith, Miss Ada, 337
Smith, Miss Amelia, 379
Smith, Miss Hilda, 204
Smith, Mr. A. P. S., 218
Somme, The, 461
South Africa, Influenza Epidemic In, 305
Southampton County Council, 173
Southport War Hospital, 315
Southwark Board of Guardians, 5, 27, 65, 195, 218, 235, 350
Sparring Match, A Hospital, 350
Spurling, Mrs. J. C., 524
Squares, The Future of the, 217
Staincliffe Poor-Law Institution, 36
Standard Ships and Food Inspection, 105
Stanley, Sir Arthur, 127, Feb. 15, viii
Stansfeld, Miss, 34
State Children's Association, 18, 359
State Medical Service, A Vision of, 109, 133, 197, 367, 373
Statistics: Nurses, 550
Stevenson, Mr. W., 26
Stewart, Miss K. S., 74
Stocking, The Empty, 282

Stonehenge, 26
Stonehouse Board of Guardians, 400
Strikes, 430, 457, 488
Stroud Nursing Association, 502
Struggle to Rise, The, 486
Subsidies, Voluntary Hospitals and, 511
Suicide of a Hospital Surgeon, 328
Surgery: The Public and "Marvellous Surgery," 441
Surgical Requisites Association, 128
Swedish War Hospital, 490
Swiney Prize, 367
Syphilis (*see* Venereal Diseases)

Taunton Red Cross Hospital, 500
Tasmania: The Doctors' Strike, 554
Taxing Donations to Hospitals, 440
Tea Adulteration, 337
Tehidy Sanatorium, 514
Temporary *versus* Permanent Hospitals, 419
Territorial Force Nursing Service, 17, 98, 144
Tetanus, The Prevention of, 195, Its Treatment, 491
Tethelin, 418
Textiles for Metropolitan Institutions, 235
Theme for Thomas Hardy, A, 304
*Third London Hospital Gazette*, 173
Third London General Hospital, 318
Thomas, Sir Lynn, 301
Thomson, Sir Courtland, 173
Three Towns Nursing Association, 380
Tobacco, Cancer of the Tongue and, 324; and Clear Thinking, 330
Torrance, Miss R., 33
Town Clerks, The Salaries of, 539
Town Planning, Ideals In, 266
Trade Unionism and the Medical Profession, 464, 482
Trade Unions and Nurses, 498
Training Schools: Nurses', 73, 97; Bringing Into Line, 571
Transfusion of Blood, Nov. 23, vi
Transvaal Inspector of Hospitals, 468
Trench Fever, 353
Tritton, Sir Ernest, 279
Tropical Diseases, New Hospital, 531
Trouville Military Hospital, 512
**TUBERCULOSIS:** The Abuse of Sanatoria by the Military, 27; Treatment of Advanced Cases, 45; Hospital Treatment, 59; Herefordshire Insurance Committee and After-Care, 65; Anti-Tuberculosis Propaganda in France, 106; Food Rations in Sanatoria, 106; Pensions, 115, 129; Ex-Service Men, 122; New Midland Sanatorium, 128; Leased Premises, 129; In the Army, 146; Peel Hall Sanatorium, 151; Epidemiology, 177; Consumptive School Children, 218; Sanatoria Shortage, 219; Wages for Colony Patients, 220; New Farm Colony, 235; The Tuberculosis Health Visitor, 243; Hospital and the Law, 280; Provision for Children, 281; Discharged Soldiers, 303; Institutional Treatment, 306; Army Nursing Sisters and Officers, 314; Sheffield Institution, 328; Topics, 461; Hostel for London, 420; Boarding Out, 422; Functions and Limitations of Sanatoria, 425; Surgical Tuberculosis in the West, 514; Bournemouth, 526; Germany, 528, Ex-Soldiers, 530; Model Care Committee, Supp. March 22; Notts County Council, 562; Miners, 564; Cutaneous Tuberculosis, 565
Twilight Sleep, 440, 443
Tynemouth Poor-Law Hospital, 432
Types of British Nursing, Some Hospital, 166, 338

Ugly, The Cult of the, 516
Undertakers, The Discharge of, 235
Uniform, Its Uses and Abuses, 550
**UNITED STATES:** French Commissions for American Women Doctors, 25; American Red Cross, 33, 204, 215, 328, 337, 379; *American Red Cross Bulletin*, 46, 88, 130; The King's Speech to Soldiers, 63; The American St. Dunstan's, 64; The Dispensary, 95; American Hospital in Wales, 107; American Emigrants from Wales, 128; Sailors Sleeping in the Law Courts, 234; Statistics of Red Cross Work, 328; War Nurses, 373, 457; Prohibition, 403; Disabled Men, 465; Highfield Hospital, 468; Venereal Clinics, 472; Diagnostic Hospital in New York, 559
Universities and Parliament, The, 171

**V.A.D.**, 56, 119, 144, 173, 206; Training after the War; Health Visitors and Welfare Workers, 269; Scholarship Scheme, 269, 378, 500; Demobilisation in Ireland, 312; Medals for, 340; General Service Section, 364; Ministry of Pensions, 398; Work in Durham and Birmingham, 431
Vaughan, Col. Bruce, 234, 396
**VENEREAL DISEASES**, 70, 146, 184, 215, 277, 307, 309, 347, 467, 471, 472
Venice, Antwerp, Bruges—and Cardiff? 193
Vickers, Messrs., 26
Victoria League Club, 99
Victory Ball (*see* College of Nursing)
Vienna: Children's Hospital, 268
Vigano, Signorina Giuseppino, 88
Village Life and Industries, 82, 426
*Vindictive*, Relics of the, 215
Vocation or Profession, 357
Voelcker Dr. A. F., 48
Voluntary Hospital, The Drawbacks of the, 234
Voluntary War Workers, The Fate of, 312
Waldo, Dr. F. J., 370, 488

Walsall Victoria Nursing Institution, 502
Wandsworth Guardians, 431
War and Epidemic Diseases, 59
"War Beetle" Bed at Belfast, The, 108
War Bonuses: Boards of Guardians, Dec. 14, vi, 235; Frauds, 442
Warfare, Modern Methods of, 493
War, Influences on Medical Practice, 125
War, Many Races at the, 69, 89
War Memorials, 489, 501, 537
War Office: Evacuation of Civil Institutions, 379
Warren, Sir Henry, 275
War Savings, 54
Warding of Nurses, 120
Wassermann Reaction, The, 423
Watney, Miss C., 34
Wearham, Miss, 185
Welfare Work, 206, 269
Welsh National Memorial Association, 468
West Derby Union, 432
West Ham Guardians, 75
West Ham Infirmary, 36
Westminster Cathedral, Thanksgiving Service at, 316
Weston, Dame Agnes, 85
What Nurses Expect and Want, 274
Whipps' Cross War Hospital, 36, 164, 184
Whisky Cure for Influenza, 466
White, Sir W. H., 419
Whole-time Salaried Medical Service, A, 311
Wigmore Hall Medical Meeting, The, 401
Willesden Council, 66
Willesden War Memorial, 233, 398
Wills, Mr. H. H., 537
Wilson, Dr. T. Stacey, 369
Winters, Miss M. V., 35
"Winter's Pie, 1918," 274
Women Doctors in Military Hospitals, 146, 556
Women of Action in Past Wars, 189
Women of the Empire, Queen's Message to, 226
Women Workers, The Influence of, 529
Women Workers' World, The, 39, 59
Women's Occupation and its Effects, 124
Women's Sanitary Visitors' Association, 552
Women's Total Abstinence Union, Oct. 5, iv
Women's War Service, 142
Woodhead, Dr. W. C., 26
Woolcock, Mr. W. J. U., 302
Woolwich: Death Rate, 436
Worcester, Sectional Subscriptions at, 216; City and County Nursing Association, 432
Worst War of the World, The, 131, 263
Wound Pension and Taxation, 217
Wrong Spirit, The, 395
Wyndham, Sir C., 340

Years that Lie Ahead, The, 266
York City Asylum, 151
Young Men's Christian Association, 5
Young Women's Christian Association, 379

PRINTED BY

SPOTTISWOODE, BALLANTYNE AND CO. LTD.

LONDON, COLCHESTER AND ETON.

### SOME YEAR-BOOKS.

The Medical Annual: A Year-book of Treatment and Practitioners' Index. (John Wright and Sons, Ltd. 1918. Price 10s. net.)

The thirty-sixth issue of this annual contains, in addition to the usual classified information, reviews of therapeutic and medical and surgical progress during 1917. The dictionary of treatment embraces 560 pages of matter valuable to the practitioner, and much of it of especial assistance to those who are called upon to treat patients with diseases and injuries attributable to warfare.

The Medical Register. (Published for the General Medical Council by Constable and Co., Ltd. 1918.)

The Register contains the usual legal and tabular features. The foreign list of practitioners now extends to four pages, as against slightly over one page before the war.

The Dentists' Register. (Published for the General Medical Council by Constable and Co., Ltd. 1918.)

The usual information is presented in the familiar form.

